# Management of Severe Facial Nerve Cross Stimulation by Cochlear Implant Replacement to Change Pulse Shape and Grounding Configuration: A Case-series

**DOI:** 10.1097/MAO.0000000000003493

**Published:** 2022-01-27

**Authors:** Susan T. Eitutis, Robert P. Carlyon, Yu Chuen Tam, Marina Salorio-Corbetto, Zebunnisa Vanat, Karen Tebbutt, Rhian Bardsley, Harry R. F. Powell, Shibasis Chowdhury, James R. Tysome, Manohar L. Bance

**Affiliations:** ∗ Emmeline Centre, Cambridge University Hospitals NHS Foundation Trust; † Cambridge Hearing Group, Department of Clinical Neurosciences, Cambridge Biomedical Campus, University of Cambridge; ‡ Cambridge Hearing Group, MRC Cognition & Brain Sciences Unit, University of Cambridge, Cambridge; § Guy's and St. Thomas’ NHS Foundation Trust, London; || Oticon Medical, UK

**Keywords:** Biphasic anodic stimulation, Cochlear implant, Distributed all polar grounding, Facial nerve stimulation, Programming, Pseudo-monophasic, Reimplantation

## Abstract

To investigate the combined effect of changing pulse shape and grounding configuration to manage facial nerve stimulation (FNS) in cochlear implant (CI) recipients.

**Patients:**

Three adult CI recipients with severe FNS were offered a replacement implant when standard stimulation strategies and programming adjustments did not resolve symptoms. Our hypothesis was that the facial nerve was less likely to be activated when using anodic pulses with “*mixed-mode*” intra-cochlear and extra-cochlear current return.

**Intervention:**

All patients were reimplanted with an implant that uses a pseudo-monophasic anodic pulse shape, with mixed-mode grounding (stimulus mixed-mode anodic)—the Neuro Zti CI (Oticon Medical). This device also allows measurements of neural function and loudness with monopolar, symmetric biphasic pulses (stimulus MB), the clinical standard used by most CIs as a comparison.

**Main Outcome Measures:**

The combined effect of pulse shape and grounding configuration on FNS was monitored during surgery. Following CI activation, FNS symptoms and performance with the Neuro Zti implant were compared with outcomes before reimplantation.

**Results:**

FNS could only be recorded using stimulus MB for all patients. In clinical use, all patients reported reduced FNS and showed an improvement in Bamford-Kowal-Bench sentences recognition compared with immediately before reimplantation. Bamford-Kowal-Bench scores with a male speaker were lower compared with measurements taken before the onset of severe FNS for patients 1 and 2.

**Conclusions:**

In patients where CI auditory performance was severely limited by FNS, charge-balanced pseudo-monophasic stimulation mode with a mixed-mode grounding configuration limited FNS and improved loudness percept compared with standard biphasic stimulation with monopolar grounding.

Cochlear implants (CIs) provide sound perception using a series of electric pulses to stimulate the auditory nerve (AN). However, 5.7% of patients (range, 0.7–43%) will also experience unwanted facial nerve stimulation (FNS) (1–3). FNS can begin at CI activation, or may take >10 years to manifest (4). Although the physiological mechanisms of FNS are unclear, patients with conditions such as otosclerosis, ossification, cochlear abnormalities, and with lateral wall arrays are more likely to experience FNS (1,4–7).

FNS is typically managed with programming modifications (1), such as increasing pulse duration (PD) to reduce pulse amplitude (8,9), reducing comfort levels, using triphasic pulses or deactivating electrodes causing FNS (10,11). However, these interventions may also lower auditory performance (8,12,13). In extreme cases, this may necessitate reimplantation (8,13). In such instances, programming parameters including pulse shape, grounding configuration, and loudness coding are essential to consider as these may affect facial nerve (FN) activation.

Most CIs use cathodic-leading symmetric biphasic pulses. These are likely an inefficient way of stimulating the auditory system because the two opposite-polarity pulses are close in time and partially cancel each other at the nerve membrane, and because the human AN is more sensitive to anodic than cathodic stimulation (14–18). MED-EL have exploited this polarity sensitivity by using anodic-dominated triphasic pulses to manage FNS (Fig. 1) (19,20), on the (implicit) assumption that the FN has different polarity sensitivity than the AN (19,20). However, triphasic shapes may be suboptimal for reducing FNS because they require more current to reach most comfortable level (MCL) compared with symmetric biphasic pulses (19).

**FIG. 1:**
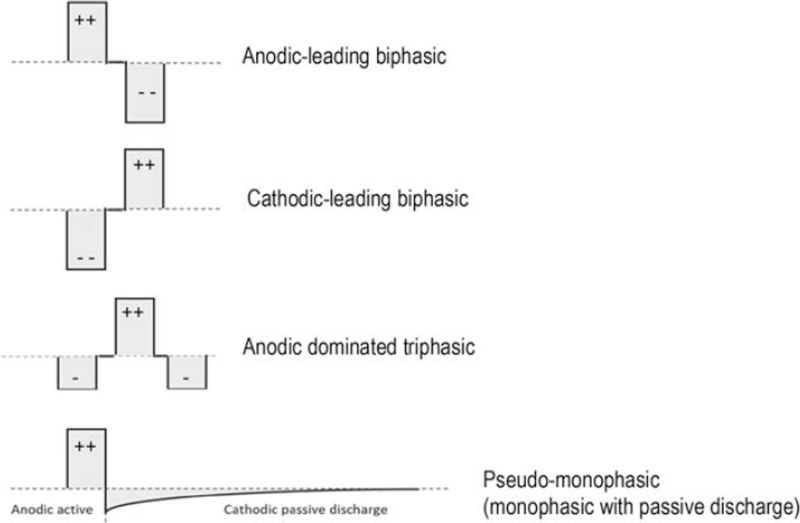
**Schematic of pulse shapes.** The top image shows the pulse type used in monopolar biphasic (Stimulus MB) stimulation and the bottom image shows the pulse type used in mixed-mode anodic (Stimulus MMA) stimulation in the Oticon implant. Biphasic pulses, as shown by the top two schematics, can either be anodic-leading or cathodic-leading. The anodic-leading pulses are used during electrically evoked compound action potential (eCAP) measurements with the Oticon Medical cochlear implant, while cathodic-leading pulses are the industry standard for biphasic stimulation. Biphasic stimulation is often used with monopolar grounding, although, when applied with bipolar grounding (intra-cochlear current return), the return electrode will have an opposite phase to the stimulation electrode. In such cases, we are unable to differentiate if the stimulating or ground electrodes are responsible for FNS. Triphasic pulses are clinically available for MED-EL devices, as shown in the third schematic. These triphasic pulses are anodic dominated, with a large central anodic phase that is charge balanced by two cathodic pulses, each 50% of the total charge of the large anodic phase. Pseudo-monophasic stimulation used in the Oticon implant uses an anodic pulse with passive discharge. Here, the maximum amplitude of the sharp cathodic phase is 20% of the anodic pulse amplitude. Timing characteristics of the passive recovery depend on the electrode impedance and fixed blocking capacitor, where for an average MAP passive discharge takes 2 to 3 ms to achieve charge balance.

FNS can also be influenced by the current path from stimulating to ground electrode. Most devices use monopolar stimulation, resulting in wide electric fields (21,22). In contrast, intra-cochlear current return paths, such as bipolar or tripolar stimulation, have smaller spatial electric fields but at the expense of less stimulation at the AN (22). Therefore, reduction of FNS may also be possible by modifying grounding configurations.

Finally, FNS may be affected by PD. Increasing PD reduces the pulse amplitude (current) needed to excite a single AN fiber or to produce a given loudness (23,24), and the relative effects of these two parameters depend on the integration time constant of the neurone cell membrane (25). Clinical advice for management of FNS often includes increasing PD and reducing pulse amplitude, which makes the (often implicit) assumption that increasing PD has a smaller effect on FN than on AN activation.

The Neuro Zti CI (Oticon Medical) was of interest to us for managing severe FNS, because it offers a unique simulation approach. It adjusts loudness by PD instead of amplitude, so that loud stimuli are encoded by long PDs rather than high current levels, potentially limiting FN activation. Furthermore, for clinical stimulation it uses anodic monophasic pulses, with charge being recovered relatively using passive capacitive discharge (Fig. 1) (26); approximating the pseudo-monophasic pulses widely used in studies of polarity sensitivity (14–18). These studies have shown that at equal stimulation levels pseudo-monphasic pulses (anodic:cathodic passive recovery duration ratio of eight) had higher loudness ratings compared with triphasic pulses (anodic:cathodic duration ratio of two) (14,15,18). Hence, the advantage of pseudo-monophasic pulses for reducing stimulation levels will depend on the “effective” duration ratio produced by the capacitive discharge implemented for the Neuro Zti device.

The Neuro Zti also applies a “mixed-mode” grounding configuration, which retrieves about 80% of the injected charge via all nonstimulating intra-cochlear electrodes, with the remaining roughly 20% returned by the extra-cochlear ground (27). Hereafter, we refer to mixed-mode, anodic pseudo-monophasic stimulation type as “mixed-mode anodic (MMA),” and the more classic monopolar symmetric biphasic pulse as “MB.”

We hypothesize that this combination of pulse shape, grounding changes, and loudness coding may contribute to a reduction in FNS. Here we compare MMA and MB stimulation as strategies for managing FNS in the same patient, thereby controlling for nonspecific consequences of reimplantation such as a period of nonstimulation between the operation and implant activation. Since FNS management may limit audibility, the loudness of each stimulus was tested to ensure audibility was maintained. This is important because the goal is not simply to minimize FNS but to do so while reaching MCL. The present study was officially registered as an audit of a new therapeutic indication in our institution, and does not require ethics approval for this use.

## CASE SERIES

We report three adult CI recipients with severe FNS which could not be managed with programming changes. FNS began between initial activation to after 13-years of CI use (Table 1). Programming to minimize FNS resulted in insufficient loudness and limited auditory benefit. All devices were confirmed to function within normal limits with manufacturer performing integrity testing.

**TABLE 1:** Patient and cochlear implant details before reimplant surgery

Pt	Aetiology	Implant, Yr	Start of FNS^*a*^	Electrodes With FNS^*b*^	Grounding Modes Tried^*e*^	Pulse Types Tried	Integrity Test	Break Given
1	Unknown, progressive	ConcertoFlex28, 2012	2 mos^*c*^	12/12	Monopolar	BiphasicTriphasic	Yes	No
2	Meningitis	CI22M, 1995	13 yrs^*c*^	22/22	Bipolar + 3Common groundPseud-monopolar^*d*^	Biphasic	Yes	Yes, no improvement (1 wk)
3	Ototoxicity, with possible dehiscence	SynchronyFlex28, 2019	Switch-on	10/12 (2 extra-cochlear)	Monopolar	BiphasicTriphasic	Yes	No, FNS since switch-on

a Start of FNS in months (mos) or years (yrs) following initial activation (switch-on).

b Electrodes with FNS immediately before reimplantation.

c FNS initially managed with programming, but continued to progress.

d Refers to using a single intra-cochlear electrode as the ground for all active electrodes (typically electrode 1).

e 22 M device can only be programmed with intra-cochlear return strategies, monopolar grounding is not an option for this implant however is available in newer Cochlear Corporation devices.

**FIG. 2:**
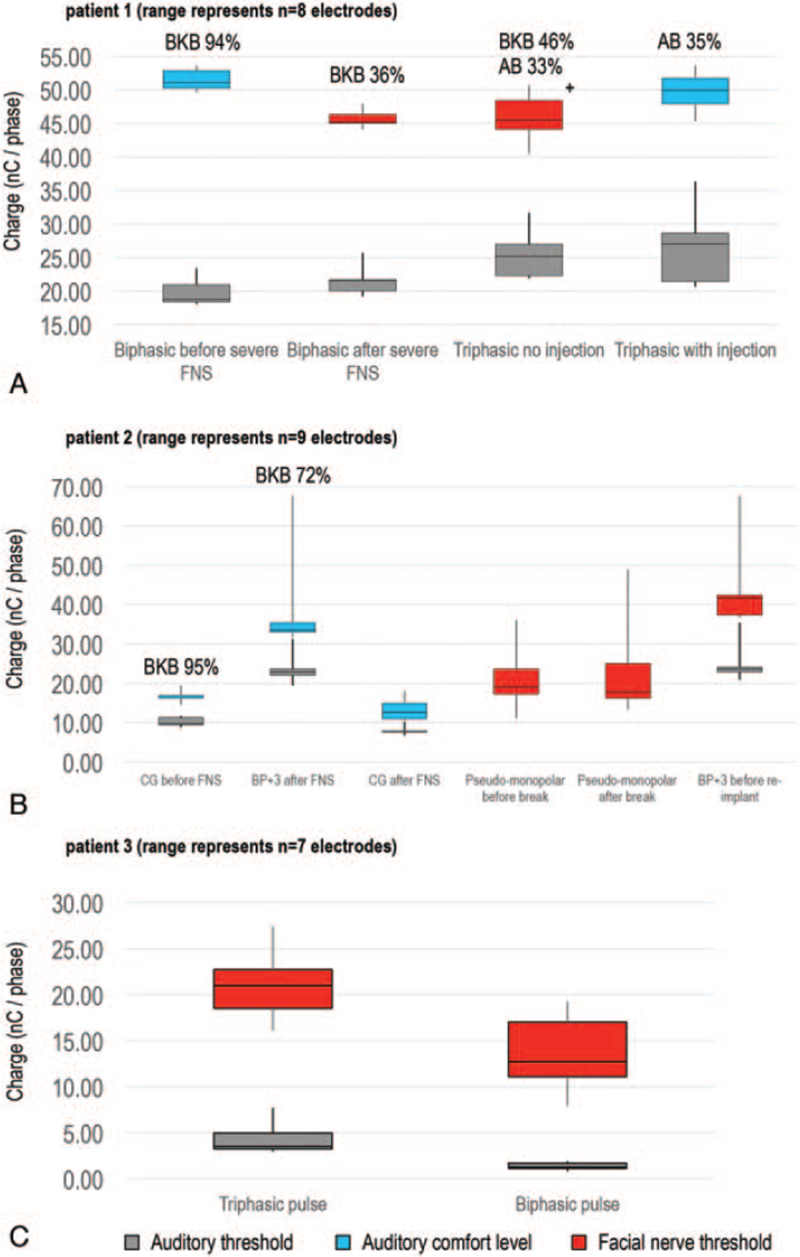
Comparison of charge range for threshold and comfort levels when adjusting pulse shape for patients 1 and 3, or grounding configuration for patient 2. Auditory thresholds (dark grey), auditory comfort thresholds (blue), and facial nerve (FN) thresholds (red). Injection refers to induced temporary paralysis of facial nerve. Levels are shown in charge (nano Colombs per phase (nC/phase)) provide a consistent reporting metric between companies, and accounts for differences in proprietary clinical units and pulse width. *A*, Patient 1: FN thresholds where stimulating levels were reported to have a “soft” loudness level. Box plots represent the range of charge across electrodes 1, 2, 3, 4, 7, 8, 10, and 11 (n = 8) for all conditions. *B*, Patient 2: FN thresholds where stimulating levels were reported to have “moderate-loud” loudness level. Box plots represent the range of charge across electrodes 3,4,6,7,8,9,15,17, and 19 (n = 9) for all conditions. *C*, Patient 3: FN thresholds where stimulating levels were reported to have “soft” loudness level. Box plots represent the range of charge across electrodes 1, 4, 5, 6, 7, 8, and 9 (n = 7) for all conditions. For all patients, measurements were taken for active electrodes only, and were not included for electrodes that had to be deactivated due to severe FN stimulation. FN thresholds only were measured for pseudo-monopolar settings, auditory thresholds were not measured as no active MAP was provided using this grounding configuration. Speech scores with a male speaker (Bamford-Kowal-Bench [BKB] sentences or Arthur-Boothroyd [AB] words) are shown above each condition. + FN responses were measured on five of eight electrodes, with the remaining three set to a reported “soft” loudness to provide an equal volume across all active electrodes. BP + 3 indicates bipolar +3; CG, common ground; FNS, facial nerve stimulation; pseudo-monopolar, single intra-cochlear ground for all electrodes.

*Patient 1* was implanted in 2012 with the MED-EL Concerto Flex28 device. They developed FNS during their first year of CI use. Initial speech scores were good without lip-reading, but declined as FNS worsened. Reported loudness was limited to “soft,” with similar FNS thresholds when using biphasic and triphasic stimulation. However, auditory thresholds were higher for triphasic pulses (Fig. 2A). To determine if stimulation levels were constrained by FNS, or if AN function had degenerated, the FN eye branches (most affected) were temporarily paralyzed with local anesthetic. With this, adequate loudness levels were achieved without FNS, while auditory thresholds remained unchanged. This indicated that the AN was responsive to higher levels of stimulation, thus the decision to trial the Neuro Zti device was made.

*Patient 2* was implanted in 1995 with the Cochlear CI22 M electrode. They developed FNS after 13 years of CI use. Speech recognition without lip-reading was initially excellent, but performance deteriorated following FNS onset. Current requirements for auditory and FN thresholds were different across grounding configurations (Fig. 2B). However, FNS remained problematic in all conditions despite intra-cochlear grounding strategies. Due to the late onset of FNS, and changes to auditory thresholds over time, a short break was advised, with the hope that resting the AN might reduce the current needed for auditory function, and so improve FNS. However, the FN threshold and perceived loudness did not change; therefore, reimplantation with the Neuro Zti device was offered.

*Patient 3* was implanted in August 2019 and experienced FNS on activation of their MED-EL Synchrony Flex28 device. They were never able to understand speech with their CI alone. Programming alterations did not improve FNS or benefit hearing, despite trialling biphasic and triphasic stimulation (Fig. 2C). Therefore, reimplantation with the Neuro Zti CI was offered.

## METHOD

### Implantation

All patients were reimplanted with the Neuro Zti^*EVO*^ electrode using a round window approach.

### Stimuli

Two stimuli were tested with the Neuro Zti device using Genie Medical CI (GMCI) clinical software version 1.2 (Fig. 1). Parameters for stimulation in GMCI include stimulus duration (SD, μs), stimulus amplitude (SA, μA), and stimulus level (SL, nC/phase) (supplementary table 1). Results were converted to charge (nano-Coulombs / phase; nC/phase) to enable comparison of overall stimulation level between stimulus types and implant companies. This conversion, however, does not address the impact of different stimulation rates, pulse shapes, and/or electrode locations.

1. Stimulus MB can only be accessed using the “electrically evoked compound action potential” (eCAPs) measurement in GMCI. Stimulus MB is similar to the strategies implemented in most other CIs, except the symmetric pulse is anodic-leading in the Neuro Zti and cathodic-leading in other devices (Fig. 1). Stimulus MB is the only option for biphasic stimulation with Neuro Zti and was used to check FN thresholds during intraoperative tests and subjective loudness growth postoperatively.
2. Stimulus MMA, also called “*Biphasic Anodic with full capacitive discharge and distributed all polar grounding”* by Oticon Medical, is the standard stimulation for clinical MAPs, but can also be accessed via the “stapedius” measurement in GMCI. Stimulus MMA using the “stapedius” test was used to check FN thresholds during surgery and subjective loudness growth postoperatively.

Comparing MB and MMA stimuli allowed for a within-subject comparison of the effect of pulse shape and grounding configuration combinations on FNS using the same implant. Additionally, intraoperative checks with the anodic-leading stimulus MB served as a baseline comparator to cathodic-leading biphasic stimulation and to control for the possible improvements of FNS being due solely to the effects of implant nonuse between surgery and new-implant activation.

### Intraoperative Measures: Comparing FN Thresholds Between Stimuli

The charge required to generate a FN response was measured first with stimulus MB and then stimulus MMA. The presence of a FN response was determined using the FN monitor (MedTronic NIM response 3.0) with threshold set at 25 μV. Stimuli were presented at 80 pulses per second (pps) with SD of 30 μs and increased in step sizes of ∼ 5 nC/phase until a FN response was measured, or to a maximum 70 nC/phase. Once an FN response was detected, no further increases to stimulus level were made. Measurements were taken on five electrodes for patient 1, and on all electrodes for patients 2 and 3.

Finally, FNS was checked with stimulus MMA on “live mode,” where threshold and comfort levels were set to 55 nC/phase and 80 nC/phase respectively for all electrodes, using 8 maxima at 500 pps.

### Postoperative Testing: Comparing Loudness Growth Between Stimuli

Patients rated the loudness of stimulus MB and MMA using a loudness rating scale. The level of each stimulus was increased until the patients reported a “loud” level or until FNS occurred. Four measurements were taken for each electrode in the order: Stimulus MMA, MB, MMA, MB. If FNS was reported, no further increase to stimulation levels was made and a 15-minute break was given to minimize any change in sensitivity of the FN before resuming testing.

Stimulus MB parameters were set to 8 averages with SD of 30 μs (supplementary figure 1). Stimulation levels were started just below auditory threshold (10–15 SA) and increased by 2 SA until a loud level was reached, after which ascending and descending steps of 1 SA were used to find the most comfortable listening level. Stimulus MB could only be presented using the eCAP setting, which implements the forward masking paradigm, where “probe only” stimulation lasts 2.5 seconds.

Stimulus MMA was tested by adjusting the SA as described above, with rate set at 80 frames/s and SD at 30 μs (supplementary figure 2). Each stimulus was presented in 2 to 3 seconds bursts to match the 2.5 seconds “probe only” presentation of stimulus MB (as determined by the eCAP measurement).

### Postoperative Testing: Routine Testing

Following CI activation, reports of FNS were monitored and loudness levels were measured for all active electrodes. Speech recognition was tested using recorded Bamford-Kowal-Bench (BKB) sentences or Arthur-Boothroyd words.

## RESULTS

### Intraoperative Measures

All patients had intraoperative testing completed (Table 2). All patients used their original implant consistently until the day of surgery; therefore, there had been no “resting of the AN” at the time of intraoperative testing.

**TABLE 2:** Intraoperative and postoperative details for reimplanted device

		FNS Intraop^*a*^		Loudness Growth Postop^*a*^				
Pt	Full Insertion (Yr)	Stim MB	Stim MMA	Stim MB	Stim MMA	FNS on Active MAPs	Pre-reimplantation Speech Tests (Yr)^*b*^	Speech Tests Post Reimplantation
1	2 out, (2019)	Yes, on 12/16 electrodes tested	No	FNS at “soft” levels	Unable to reach “loud” even at max output, no FNS	No	- 94% BKB MQ (2014)- 25-46% BKB MQ, 0% BKB FQ (2018)- Required written communication (2019)	**1-yr**- 80% live-voice open-set sentences- 36% BKB MQ22% BKB FQ**1.5 yrs**- 50% BKB MQ- 70% BKB FQ
2	7 out at surgery, 10 at switch-on (2020)	Yes, on 1/14 electrodes tested	No	Good, no FNS	Good, no FNS	No	- 95% BKB MQ, 99% BKB FQ (2014)- 72% BKB MQ, 72% BKB FQ (2019)- Relied on lip reading (2020)	**7 wks**- 50% BKB MQ- 78% BKB FQ
3	Yes (2020)	Yes, on 20/20 electrodes	No	FNS below threshold	Good, no FNS	No	Unable to reach sufficient volume for speech understanding	**4 mo**- 62% BKB MQ- 24% BKB FQ- 78% CUNY

a Testing completed on intra-cochlear electrodes only.

b Speech testing reported as Bamford-Kowal-Bench (BKB) sentence scores when available for male speaker in quiet (MQ) and female speaker in quiet (FQ) or as City University of New York Sentences (CUNY). MB indicates monopolar biphasic; MMA, mixed mode anodic.

Patient 1 had a fully inserted MED-EL implant, but resistance during reimplant resulted in two extra-cochlear electrodes. Patient 2 had a fully inserted CI22 M device, and the Neuro Zti implant was inserted to the same depth as the original device. However, the *EVO* electrode was longer than the CI22 M, leaving seven extra-cochlear electrodes. Patient 3 had a partially inserted MED-EL device, but fully inserted Neuro Zti implant.

No FNS was observed in any of the three patients with stimulus MMA on stapedius or live mode. For stimulus MB, patient 1 had FNS on 12 of 16 electrodes. Extra-cochlear electrodes were not tested, nor were electrodes three or five due to time constraints. Following surgery for patient 1, we found with additional testing that stimulation artifact from the CI could elicit a FN monitor response. Although movement in the cheek was observed during testing, FN thresholds for patient 1 may not accurately reflect myogenic responses as stimulus artifact may have triggered an audible alert from the FN monitor. Subsequently, traces were examined directly on the FN monitor, looking for the typical “broad” FN response rather than relying on audio alerts (20). Waveforms for patients 2 and 3 were therefore monitored to exclude stimulation artifacts and FNS activation was confirmed by observing/palpating for facial movement.

Patient 2 had no FNS with stimulus MB on any of the 13 electrodes tested when the FN monitor threshold was set to 25 μV, but at 20 μV, a FN response was recorded on electrode 16. Patient 3 had FNS on all electrodes. The stimulation level at which FNS was elicited for each patient can be found in Table 3.

**TABLE 3:** Thresholds for FNS during reimplant surgery using monopolar biphasic stimulation.

Electrode	20	19	18	17	16	15	14	13	12	11	10	9	8	7	6	5	4	3	2	1
Patient 1: FN threshold	47	33	40	40	37	33	x	47	x	40	47	x	47	40	47		x		EC	EC
∗Patient 2: FN threshold	x	x	x	x	47	x	x	x	x	x	x	x	x	EC	EC	EC	EC	EC	EC	EC
Patient 3: FN threshold	13	13	13	13	13	13	13	13	13	13	13	13	13	13	20	13	27	33	33	47

Facial nerve (FN) thresholds are reported as the charge required (nC/phase) for stimulation of the FN when the FN monitor was set to 25 μV. ^*a*^FN monitor threshold reduced from 25 to 20 μV. EC indicates extra-cochlear; x, no FNS; “Blank”—not measured.

### CI Programming

CI activation occurred 2 to 6 weeks after reimplant surgery. Electrode position was confirmed using postoperative X-ray. Extra-cochlear electrodes were deactivated for patient 1 (2 of 20 electrodes) and patient 2 (10 of 20 electrodes—while only 7 electrodes were extra-cochlear during surgery, 3 more appear to have extruded postimplantation). Additionally, five more electrodes were deactivated for patient 1 due to poor auditory perception including no audible sound, elevated thresholds or poor loudness growth, not because of FNS. Patient 3 had a full insertion and did not require any electrodes to be deactivated.

All patients were programmed with the default rate of 500 pps. MCL could be reached for all active electrodes during routine programming (stimulus MMA). Minimal changes were required during the first 2 months for all patients. For patient 1, settings have remained stable with no reported FNS >1.5 years postactivation. To date, no FNS has been reported for any of the patients with their clinical MAPs.

### Postoperative Loudness Growth

Loudness growth was measured on 1 electrode for patient 1 (1.5-yr visit), 2 electrodes for patient 2 (7-wk visit), and 3 electrodes for patient 3 (2-mo visit) (Table 2). Patient 1 experienced FNS between 22 and 24 nC/phase in addition to a “soft” auditory percept with stimulus MB. This was below FNS thresholds measured during reimplant surgery (33–47 nC/phase), and below FNS thresholds with their MED-EL implant (44–48 nC/phase). They did not experience any FNS with stimulus MMA, but only reached “medium” loudness, even at the maximum output (60 nC/phase). This limited loudness could arise from compliance limitations of the device, poor loudness growth at high stimulation levels, or the effect of very low pulse rate on perceived loudness when testing a single electrode (28,29). However, additional calculations ruled out compliance as the cause of loudness plateaued.

Patient 2 did not experience FNS during testing for either stimulus type, and a “loud” sound percept was reached between 23 to 25 nC/phase for MMA and 15 to 17 nC/phase for MB stimuli. Patient 3 experienced FNS below auditory threshold when tested with stimulus MB; however, “loud” sound levels could be reached between 27and 36 nC/phase with MMA stimuli.

### Functional Outcomes

Immediately before reimplant surgery, all patients relied on lip-reading and/or written communication because of severe FNS. All patients reported a subjective improvement in communication with the new implant, and could follow conversation in a quiet room, without lip reading, and with minimal repetition. Patients 1 and 2 had difficulty adapting to the new sound after long-term CI use, but continue to report acclimatization over time. Patient 3, who had limited CI experience before reimplantation, reported hearing lots of environmental sounds but a lack of speech clarity 3-months postactivation.

Functional testing was completed for all patients (Table 2). Patient 1 showed improved speech recognition for male and female speakers compared with the most recent results with their old implant. Patient 2 showed similar BKB outcomes with a female speaker compared with their previous implant when they still had auditory benefit, but BKB scores with the male speaker were lower with the new device. Patient 3 could complete speech testing for the first time with a CI. Although speech scores for both long-term CI users (patients 1 and 2) improved compared with immediately before reimplantation, their best functional outcomes were measured with their original implants before the onset of FNS.

## DISCUSSION

These cases demonstrate that CI reimplantation to change both pulse shape and grounding configuration can successfully manage severe FNS after routine programming had failed. All patients achieved adequate volume percept without FNS for stimulus MMA, which was not possible when changing pulse shape (MED-EL) or intra-cochlear grounding strategies (Cochlear). This may be due to the greater sensitivity of the human AN to anodic current (14,15)—and that the FN does not share this polarity sensitivity to an equal measure (19,20) —and/or because of the use of mixed-mode grounding.

### Programming Modifications

Reimplantation for the treatment of FNS is uncommon as most incidents of FNS can be managed with programming. In the most difficult FNS cases, management may include changing pulse shape or grounding configuration when adjusting PD or comfort levels is unsuccessful (20,30).

In our small case series, changing from biphasic to triphasic pulses with monopolar stimulation showed no difference in FNS thresholds for patient 1 (Fig. 2A). Sufficient loudness levels using triphasic stimulation were reached only when the FN was temporarily deliberately paralyzed. For patient 2, changes to grounding configuration showed differences in current requirements for both auditory and FN thresholds (Fig. 2B). Despite stimulation level differences, changing grounding configuration did not improve auditory function or reduce FNS in any condition. From these independent manipulations, it is clear that changes to either the pulse type or grounding configuration can impact the overall charge required for auditory and FN stimulation.

By reimplanting with the Oticon Medical device, grounding configuration and pulse shape were both modified. Although FNS was resolved in these instances, the GMCI clinical software did not allow us to determine which of these two factors was more important. Currently, no clinical CI software allows for independent manipulation of grounding configuration and pulse shape within the same device. It may, however, be possible to disentangle these two factors and their effects on FNS in future using research software/hardware.

### Break From CI Stimulation

Late-onset FNS may sometimes be triggered when increasing auditory stimulation levels are required. Overstimulation of the AN can increase eCAP thresholds in guinea pigs (31) and lead to cellular loss at different levels of the central auditory pathway (32). If long-term overstimulation has an excitotoxic effect on the AN, it is possible that introducing a break from CI use could regain some sensitivity of the AN to stimulation. This approach has previously been successful in our clinic (unpublished data) for patients where current levels and FNS had increased over time.

For patient 2, a 1-week break was suggested when FNS developed after more than 10 years of CI stimulation, but no differences to auditory perception or FNS were found following this break. However, the short duration of this break may have been insufficient to allow the AN to recover. The extended break between surgery and CI activation may therefore be one mechanism by which the reimplant improved FNS. However, intraoperative testing still showed FN responses to electrical stimulation when using stimulus MB but not MMA in all patients. This indicates that the benefit observed could not entirely be attributed to the break between surgery and implant activation.

### Progress Following Reimplant

Following reimplant all patients showed an improvement in FNS and functional hearing compared with immediately before surgery. However, the long-term CI users have yet to return to their best performance levels. Before developing FNS, patients 1 and 2 achieved >90% on BKB sentences. Although function with the new device has gradually improved, performance remains 40-percentage point below their best scores. While some functional difficulty may be related to needing an acclimatization period, we must also consider how cochlear trauma (multiple surgeries), partially inserted implants, changes to rate, and differences in stimulation strategy affect the quality of sound. Patients 1 and 2 did not have full electrode insertion. Although a full insertion is not always necessary for speech recognition, changes to the position of the new relative to the old implant may impact the sound quality.

For patient 2, reimplantation resulted in a marginally shallower insertion compared with their CI22 M device. Due to the longer length of the Neuro Zti electrode array and some postoperative extrusion, only 10 electrodes remained in the cochlea to stimulate a region previously activated by 20 electrodes. Consequently, cochlear neural regions adapted to stimulation from several electrodes for decades, must now adjust to stimulation from a single electrode with a broader frequency range and potentially less frequency resolution. This factor, and the limited electrical stimulation in low-mid frequency regions of the cochlea, may restrict how much immediate benefit can be expected for this patient.

Additionally, default stimulation rates may not be optimal for every patient. When auditory performance was optimal, patients 1 and 2 were using stimulation rates between 400 and 600 pps and 200 and 250 pps respectively. Although before reimplantation rate changes were tried to improve FNS, further investigation with the Neuro Zti is required to determine if deviation from the default rate (500 pps) is needed to improve performance.

### Limitations

We were unable to independently test the effects of grounding configuration and pulse type for managing FNS. At this time, the GMCI clinical software does not allow for independent manipulation of these two parameters. Hence, their relative contribution needs further exploration. For example, if grounding configuration is the most important factor, reimplanting the MED-EL recipients with the Cochlear device to make use of intra-cochlear grounding strategies may have been equally or possibly more effective. Similarly, we do not know whether the pseudo-monophasic pulse shape used in stimulus MMA is more or less effective than the anodic-triphasic pulses in MED-EL devices. However, as it seems likely that both pulse shape and mode of stimulation are important (20,33), the combination of anodic stimulation with mixed-mode grounding available in the Neuro Zti implant would make it a good choice for severe cases of FNS that warrant reimplantation.

Reimplantation due to FNS is rare and there is limited clinical evidence to support successful management of FNS by reimplanting with the same device (8,13,34). FNS may continue to be a problem even after reimplantation (34). In our center, recent evidence is only available from a single “control sample.” This patient was reimplanted with the same device and showed improvement of FNS at 10 months following reimplantation, but continued to experience nonauditory sensations (primarily pain) with their new implant, and functional performance is worse than pre-FNS levels (see supplementary table 2). Further investigation is certainly required to compare the success of reimplantation to manage FNS with like-for-like replacements verses changing to the Neuro Zti device.

## CONCLUSION

The case series presented here suggests that reimplantation with the Neuro Zti device may be an effective strategy for managing severe FNS that cannot be resolved by standard programming techniques. So far, none of the presented patients have experienced FNS since reimplantation. However, these results only reflect ≤1.5 years of CI use. Due to the possibility of delayed FNS onset, longitudinal monitoring will be essential to fully understand the stability of this intervention.

## Supplementary Material
