# Supplementary material for: Management of Severe Facial Nerve Cross Stimulation by Cochlear Implant Replacement to Change Pulse Shape and Grounding Configuration: A Case-series
Source: Otol Neurotol. 2022 Jan 27;43(4):452–9. doi: 10.1097/MAO.0000000000003493 (PMC8915992; doi:10.1097/MAO.0000000000003493)
Supplement: Supplementary file 1 [file mao-43-452-s001.docx]

Supplementary table 1: Clinical units and parameters for Oticon Medical Neuro Zti device

| Stimulus type | Clinical Unit | Meaning | Functional unit | Description |
| --- | --- | --- | --- | --- |
| Anodic-leading Monopolar Biphasic (MB) | SL | Stimulus Level | nC/phase (nano-Coloumbs per phase)^+^ | Level of stimulation reported in charge used when measuring electrically evoked action potentials. |
|  | SD | Stimulus Duration | µs (micro-seconds) | The length of time a pulse is played. This is a fixed parameter set by the clinician during electrically evoked compound action potential testing |
|  | pps | rate | pulses per second | The number of pulses (biphasic in this instance) played in a second, commonly referred to as pulse rate |
|  | SA | Stimulus Amplitude | µA (micro Amps) | Amplitude range, set by the clinician, that is tested during electrically evoked compound action potential testing. |
| Mixed Mode Anodic (MMA) | SD | Stimulus Duration | µs (micro-seconds) | The length of time a pulse is played. The range of values on a patient’s MAP reflects the threshold level and comfort level for each electrode. |
|  | SA | Stimulus Amplitude | µA (micro Amps) | Refers to the fixed amplitude of the anodic pseudo-monophasic pulse that is set by the clinician during programming |
|  | SL* | Stimulus level | nC/phase (nano-Coloumbs per phase)^+^ | Converted using the following formula, where 45 is a constant value:  $SL=\frac{SA*SD}{45}$ |
|  | pps | rate | pulses per second | The number of pulses (pseud-monophasic in this instance) played in a second, commonly referred to as pulse rate. Default for live MAPs is 500 pps, however this can be increased or decreased as appropriate. |
|  | Maxima |  |  | Peak picking, refers to the number of electrodes stimulated together and determined by which electrodes have the highest signal based on the incoming sound. Default for live MAPs is 8 however this can be increased or decreased as appropriate. |

+ nano Colombs per phase is a way to report the overall charge that an implant is providing. It accounts for proprietary clinical units, pulse duration (or pulse width in other companies) and pulse amplitude.

*Stimulus MMA by default is not reported as nC/phase and requires converting to charge by taking into account the threshold and comfort levels as well as the fixed amplitude set by the clinician

Supplementary table 2: Outcomes for patient re-implanted with the same as original device due to FNS

|  | Initial implant (year) | Re-implant (year) |
| --- | --- | --- |
| Aetiology | Otosclerosis |  |
| Implant | CI24M (2000)* left | CI612 (2020)* left |
| Full insertion | Fully inserted, migrated to 4 extra-cochlear | 8 extra-cochlear |
| Start of FNS | 7 years post implant | Pain at 3 weeks post re-implant |
| Electrodes with FNS | 4/22 (plus pain 22/22) | 0/22 (plus 3/22 causing pain) |
| Grounding modes tried | Monopolar  Common ground | Monopolar |
| Pulse types tried | Biphasic | Biphasic |
| Integrity test | no |  |
| Break | Clinician requested, patient declined |  |
| Speech scores** | - 92% BKB MQ (2004) – left CI only  - 26% BKB MQ (2010) – left CI only  - 96% BKB MQ (2015) – bilateral CI  - no testing with female voices completed | - 78% BKB FQ (10 months) – bilateral CI **but** continues to report poorer hearing |

*Patient bilaterally implanted with Cochlear Corporation devices, with sequential right implant in 2003

** speech testing reported as Bamford-Kowal-Bench (BKB) sentence scores when available for male speaker in quiet (MQ) and female speaker in quiet (FQ). No reported changes or measured functional decline for right implant.
