# Supplementary material for: Management of Severe Facial Nerve Cross Stimulation by Cochlear Implant Replacement to Change Pulse Shape and Grounding Configuration: A Case-series
Source: Otol Neurotol. 2022 Jan 27;43(4):452–9. doi: 10.1097/MAO.0000000000003493 (PMC8915992; doi:10.1097/MAO.0000000000003493)
Supplement: Supplementary file 2 [file mao-43-452-s002.docx]

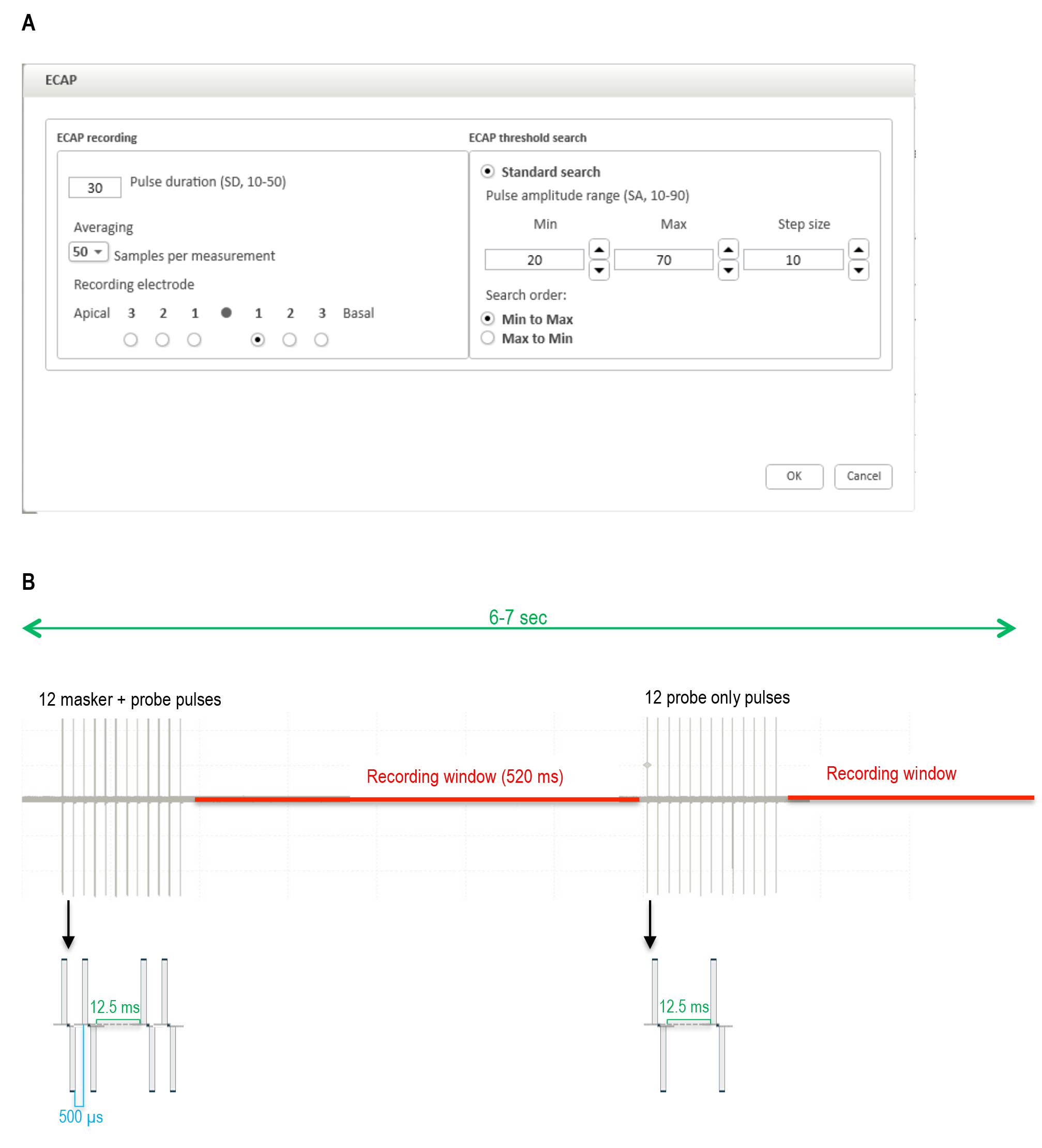


**Supplementary figure 1: Electrically evoked action potential measurements (eCAP) using anodic-leading monopolar biphasic stimulation (stimulus MB) in Genie Medical CI (GMCI).** A) setup parameter options for eCAP measurements. B) schematic of eCAP measures which use two sequences of pulse trains, the first containing 12 symmetric biphasic masker plus probe pairs, the second containing 12 symmetric biphasic pulses containing the probe alone. Each masker plus probe pair or probe only pulse is separated by a 12.5-ms gap. Total eCAP recording time is 6-7 sec, with biphasic probe only pulses being presented for a total of about 2.5 sec when using a pulse duration of 30 祍.
