# Supplementary material for: Management of Severe Facial Nerve Cross Stimulation by Cochlear Implant Replacement to Change Pulse Shape and Grounding Configuration: A Case-series
Source: Otol Neurotol. 2022 Jan 27;43(4):452–9. doi: 10.1097/MAO.0000000000003493 (PMC8915992; doi:10.1097/MAO.0000000000003493)
Supplement: Supplementary file 3 [file mao-43-452-s003.docx]

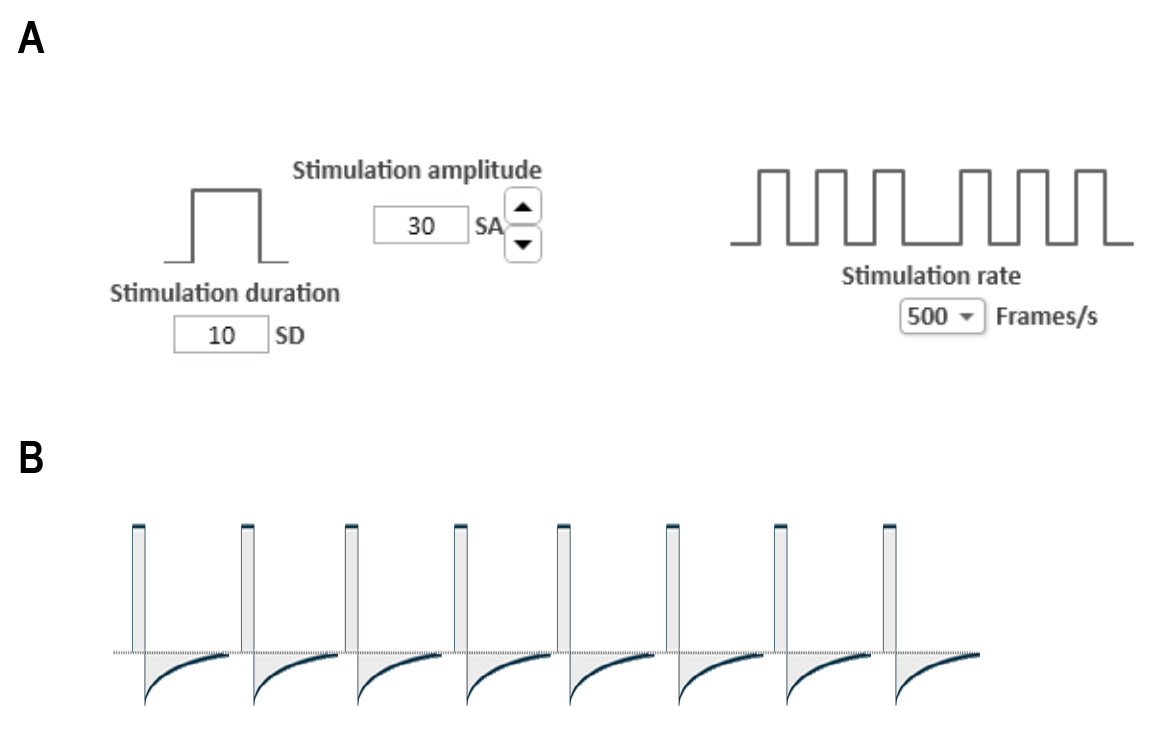


**Supplementary figure 2: Stapedius measurement using pseudo-monophasic stimulation (stimulus MMA) in Genie Medical CI (GMCI).** A) setup parameters for stapedius measurement. B) schematic of stapedius recording pulse sequence, where pulse train start and stop is controlled by the clinician.
